# Emerging misunderstood presentations of cardiovascular disease in young women

**DOI:** 10.1002/clc.23165

**Published:** 2019-03-25

**Authors:** Renee P. Bullock‐Palmer, Leslee J. Shaw, Martha Gulati

**Affiliations:** ^1^ Department of Cardiology Deborah Heart and Lung Center Browns Mills New Jersey; ^2^ Department of Radiology Weill Cornell Medical College New York New York; ^3^ Department of Cardiology University of Arizona College of Medicine Phoenix Arizona

**Keywords:** artery dissection, cardiovascular disease, emerging, ischemia, ischemic heart disease, misunderstood, mental stress, myocardial infarction, spontaneous coronary, women

## Abstract

**Background:**

Cardiovascular disease (CVD) remains the leading cause of death for females in the United States accounting for over 412 000 female deaths in 2016. CVD mortality in young women <55 years old remains significantly high and greater than that in men.

**Hypothesis:**

There is a void with regards to awareness of CVD in women. Many traditional CVD risk estimate tools fail to identify the “at risk” female and is true for the young female patient. There needs to be a shift in focus from looking for the vulnerable plaque to looking for the “at risk” patient.

**Methods:**

This review outlines the emerging misunderstood presentations of CVD in young women which include certain categories of myocardial infarction (MI) with non‐obstructive coronary arteries (MINOCA), such as spontaneous coronary artery dissection (SCAD), as well as the more stable myocardial ischemia with non‐obstructive coronary arteries (INOCA) category focusing on mental stress‐induced myocardial ischemia (MSIMI).

**Results:**

The prevalence of MINOCA in patients presenting with MI is greater in women. In younger women with CVD, SCAD is an emerging misunderstood presentation in this group of patients with type 2 SCAD being the most common form. MSIMI, a form of INOCA, is more common in women with CVD.

**Conclusions:**

There are emerging misunderstood factors that are prevalent in young women, such as SCAD and MSIMI. It is important to recognize their presentations in young women to prevent misdiagnosis, missed diagnosis as well as mismanagement of these patients to improve their clinical outcomes.

## 1 INTRODUCTION AND SIGNIFICANCE

Cardiovascular disease (CVD) remains the leading cause of death for females in the United States, accounting for over 412 000 female deaths in 2016.^1^ CVD is not just a man's disease, an equal number of women and men die from CVD each year in the United States (US)^1^). Until recently, there was a higher CVD mortality in women compared to men. Despite this, there is still a need to increase awareness of CVD in women since only approximately half of women in the United States recognize heart disease as the leading cause of death in women and is greater than that of all cancers combined.^1^ Furthermore, CVD mortality in young women (<55 years of age) remains significantly high and remains greater than that in men.^2^ The latest heart disease and stroke statistics—2019 update indicates that while there has been a decline in CVD related mortality in older female patients, there has been relative stagnation in coronary heart disease (CHD) mortality in young women over the last decade.^1^ In addition, the atherosclerotic cardiovascular disease (ASCVD) risk burden is greater in young women compared to men and the hospitalization rate for young women with ASCVD disease is lower than that in similar‐aged men.^2^ This higher mortality rate with lower hospitalization rate for young women with CVD suggests that many of these women die prior to reaching the hospital. In addition, even after treatment of their initial cardiac event, the rehospitalization rate for women with CVD disease after acute myocardial infarction (MI) is higher than that in men.^3^

There is a significant void with regards to awareness of CVD in women for both the public and also the medical profession. The presence of this void was showed by a survey performed by the Women's Heart Alliance in 2017.^4^ This study showed that only 45% of women are aware of CVD being the leading cause of death. In addition, only 22% of primary care providers and only 42% of cardiologists felt well prepared to assess the CVD risk of their female patients. There is therefore a need to educate physicians in the assessment of CVD risk in women, particularly in young women as CVD mortality in this group remains high.^4^

In addition, many of the traditional ASCVD risk estimate tools fail to identify the “at risk” female^5^ and this is particularly true for the young female patient. There needs to be a shift in focus from looking for the vulnerable plaque to looking for the “at risk” patient. In addition, there have been emerging misunderstood presentations of CVD in young women which include certain categories of MI with non‐obstructive coronary arteries (MINOCA), such as spontaneous coronary artery dissection (SCAD), as well as certain categories of ischemia with non‐obstructive coronary arteries (INOCA), such as mental stress‐induced myocardial ischemia (MSIMI) which will be outlined in this article.

## 2 EMERGING PRESENTATIONS

2.1

#### 2.1.1. Myocardial infarction with non‐obstructive coronary arteries

A novel taxonomy for young women presenting with MI was developed by Spatz et al to assist in defining the various etiologies of MIs in young women‐(variation in recovery: role of gender on outcomes of young acute myocardial infarction patients) VIRGO taxonomy.^6^ With this taxonomy, the classification for MI and prevalence according to gender is shown in Table 1. Patients with Class III and IV MI are considered as MI with non‐obstructive coronary arteries (MINOCA).

**Table 1:** The variation in recovery: role of gender on outcomes of young acute myocardial infarction patients (VIRGO) classification system: a taxonomy for young women with acute myocardial infarction and prevalence according to gender

Class	Pathology
Class I	Plaque‐mediated culprit lesion (82.5% of women; 94.9% of men)
Class II	Obstructive coronary artery disease (>50%stenosis) with:
	Class 2a supply‐demand mismatch (1.4% women; 0.9% men)
	Class 2b without supply‐demand mismatch (2.4% women; 1.1% men)
Class III	Non‐obstructive coronary artery disease (<50% stenosis) with
	Class 3a supply‐demand mismatch (4.3% women; 0.8% men)
	Class 3b without supply‐demand mismatch (7.0% women; 1.9% men)
Class IV	Other identifiable mechanism: spontaneous dissection; vasospasm; embolism (1.5% women; 0.2% men)
Class V	Undetermined classification (0.8% women; 0.2% men)

The prevalence of MINOCA in patients presenting with MI has been reported between 5.9% and 11% and occurs more commonly in women, younger patients, and non‐white patients.^7^, ^8^

**Figure 1:**
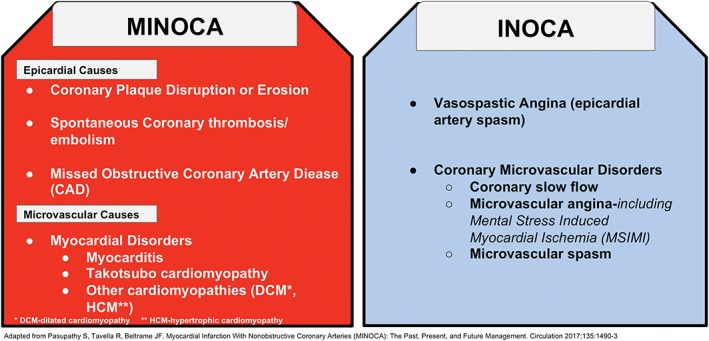
Pathophysiologic mechanisms of myocardial infarction with non‐obstructive coronary arteries (MINOCA) and ischemia with non‐obstructive coronary arteries (INOCA). CAD, coronary artery disease; DCM, dilated cardiomyopathy, HCM, hypertrophic cardiomyopathy

MINOCA should not be considered a specific diagnosis but should be considered more of a presentation with a diverse set of working diagnoses that require further evaluation to determine the specific underlying etiology in each particular case. Therefore, MINOCA patients do not have a distinguishing presentation. Underlying pathophysiological mechanisms of MINOCA can be classified as epicardial and microvascular causes as outlined in Figure 1.^9^, ^10^, ^11^ MINOCA classification continues to evolve as outlined in the European Society of Cardiology 2018 document on the definition of MI.^11^ A meta‐analysis has indicated that myocarditis has a 33% prevalence among clinical cases of MINOCA.^12^ In view of this, myocarditis will be briefly discussed.

However, in younger women with CVD, SCAD has become an emerging presentation in this group of patients and is still a somewhat misunderstood form of MINOCA and will therefore be focused on in this review.

#### 2.1.2. Myocarditis

Myocarditis is a prevalent form of MINOCA, representing a third of clinical cases of MINOCA.^11^ There are various causes of myocarditis, which include viral causes, such as parvovirus B19, coxsackie virus, human herpes virus 6, and adenovirus. Viral causes account for the majority of the cases of biopsy‐confirmed myocarditis.^11^ There are also other causes of myocarditis, such as autoimmune diseases including systemic lupus erythematosus [SLE], cardiac sarcoidosis, giant cell myocarditis, and eosinophilic myocarditis. The presentation of myocarditis may be similar to that of acute coronary syndrome (ACS) with angina, with or without ST‐segment elevation on electrocardiogram, presence of increased serum levels of troponins with the presence or absence of ventricular dysfunction in the presence of non‐obstructive coronary arteries. The pathology that underlies myocarditis resulting in this presentation rests on the presence of coronary microvascular constriction resulting in decreased myocardial perfusion. Early diagnosis of myocarditis is important as it impacts prognosis and management. Although half of these patients will have resolution of their presentation in a 2‐4 week period, up to 25% of these patients will acutely decompensate to fulminant heart failure. This is particularly true for giant cell myocarditis. Cardiac magnetic resonance imaging (CMR) should be one of the initial tests performed in cases of suspected myocarditis. CMR has been reported to be able to detect 79% of endomyocardial biopsy (EMB) proven myocarditis.^13^ The value of EMB is that it allows the underlying cause of myocarditis to be determined. Treatment of myocarditis particularly patients with ventricular dysfunction includes the use of beta blockers and angiotensin‐converting enzyme inhibitors (ACE‐I).^14^ Immunosuppression is useful in the treatment of EMB has proven infection negative autoimmune forms of myocarditis, such as giant cell myocarditis, cardiac sarcoidosis, and eosinophilic myocarditis.^11^

#### 2.1.3. Spontaneous coronary artery dissection

SCAD is defined as the non‐traumatic and non‐iatrogenic separation of the coronary arterial wall by intramural hemorrhage creating a false lumen with an intimal tear initiating the dissection or with a spontaneous bleed from the vaso vasorum. This separation can occur between the media and adventitia. The resulting hematoma compresses the arterial lumen compromising antegrade blood flow and can cause myocardial ischemia or MI. Although the dissection flap sometimes causes luminal obstruction resulting in MI, this dissection flap is not always apparent on coronary angiography and thus results in SCAD being placed under the umbrella of MINOCA. The initial prevalence of SCAD was reported as rare with less than 800 cases reported worldwide and observed in only 0.2% to 1.1% of coronary angiograms and has been reported as an infrequent cause of ACS (0.1%‐4%) and sudden cardiac death (0.4%).^15^ However, a study led by Saw et al retrospectively reviewed coronary angiograms of women aged less than 50 years old from 2009 to 2011 and it was found that SCAD is the most commonly encountered non‐atherosclerotic CAD in young women accounting for 24% of MI.^16^ Several other series have reported that SCAD is more prevalent with SCAD being the etiology for as much as 35% of ACS cases in young women aged <50 years of age.^17^ SCAD is also the most common cause of pregnancy‐associated MI accounting for as much as 43% of these cases. These findings highlight the fact that many cases of SCAD are either missed or misdiagnosed. These patients are therefore oftentimes mismanaged leading to worse outcomes. The average age of affected women ranges from 45 to 53 years of age and occurs predominantly in Caucasian women compared to other ethnic groups.^17^

There are three types of SCAD: *Type 1 SCAD* has the pathognomonic contrast dye staining of the arterial wall with multiple radiolucent lumen with or without the presence of dye hang‐up or decreased contrast clearing from the lumen. *Type 2 SCAD* is diffuse and usually involves a long segment of the vessel typically greater than 20 mm in length and usually has a smooth narrowing that can vary in severity from an inconspicuous mild stenosis to complete occlusion. There is an appreciable oftentimes subtle but abrupt decrease in arterial caliber with a demarcation from normal diameter to diffuse narrowing in which either of the following is present: (a) No response to intracoronary nitroglycerin with absence of atherosclerotic lesions in the rest of the coronary artery anatomy or (b) repeat coronary angiogram showing resolution of the dissected segment or a prior angiogram showing a normal artery or (c) intracoronary imaging with optical coherence tomography (OCT) or intravascular ultrasound (IVUS) showing an intraluminal hematoma. *Type 3 SCAD* usually mimics atherosclerotic CAD with focal or tubular stenosis with OCT or IVUS proven presence of an intra‐mural hematoma.^18^ Although the pathognomonic contrast dye staining of the coronary artery seen in type 1 SCAD is the main perceived association with SCAD, this is not the most common form of SCAD as this type accounts for only a third of SCAD cases.^15^ In fact, type 2 SCAD is the most common form and accounts for approximately 63% of SCAD cases.^15^ The most common coronary artery involved is the left anterior descending (LAD) and diagonal branches (45%‐61%), followed by the left circumflex and obtuse marginal branches (15%‐45%), then the right coronary artery (10%‐39%) with rare involvement of the left main coronary artery (4%).^17^ SCAD usually involves the mid and distal segment rather than the proximal segment which occurs in <10% of SCAD cases.^17^

The clinical features that should raise the suspicion for SCAD include the presence of particular demographic factors, predisposing conditions, associated disease states and/or triggering factors as outlined in Table 2.^15^, ^19^, ^20^ Women with SCAD present with ACS with the majority presenting as ST elevation MI (STEMI) or non‐ST elevation MI (NSTEMI), but 2% to 5% present in cardiogenic shock.^17^

**Table 2:** Demographics, predisposing conditions, associated disease states, and triggering factors that should raise suspicion for presence of spontaneous coronary artery dissection (SCAD)^15^, ^19^, ^20^

Demographics	
Age	Less than 50 years of age
Gender	Females greater than males
Ethnicity	Most common in Caucasians
Predisposing conditions	
	Lack of traditional cardiovascular disease risk factors
	Little or no evidence of typical atherosclerotic lesions
	Peripartum state
	Use of hormonal therapies, such as birth control pill use as well as use of estrogen, progesterone or testosterone. Use of B‐HCG (beta‐human chorionic gonadotropin) injections
Associated disease states	
	Fibromuscular dysplasia (FMD)
	Connective tissue diseases (Marfan's and Loetz‐Dietz syndrome, Ehler‐Danlos syndrome)
	Polycystic kidney disease
	Systemic inflammatory disorders (systemic lupus erythematosus [SLE], rheumatoid arthritis, and sarcoidosis)
	History of migraine (usually younger females, usually associated with extracoronary vascular abnormalities, such as aneurysms/pseudoaneurysms/dissection, usually associated with depression and recurrent chest pain post‐SCAD)
Triggering factors	
	Intensive exercise
	Intensive emotional stressors
	Labor and delivery
	Intense valsalva type activity
	Use of recreational drugs (cocaine, amphetamine, and methamphetamine)

An early invasive strategy with invasive coronary angiography is the recommended strategy for SCAD patients. There should be a high index of suspicion for SCAD in young women presenting with ACS as oftentimes if this diagnosis is missed and therefore mis‐managed with a non‐invasive strategy outcomes in these women are poor. Once coronary angiography is performed, if type 2 or type 3 SCAD is suspected OCT or IVUS should be performed to better define the intracoronary true/false lumen, intramural hematoma, and the site of intimal tear.^21^ OCT has a better resolution at 10 to 20 μm vs IVUS with a lower resolution of (150‐200 μm).^22^ However, IVUS has better penetration with visualization of the full vessel extent of the hematoma vs OCT with poorer penetration which limits the visualization of this full extent.^22^ Therefore, combined use of OCT and IVUS is recommended.^17^, ^22^ For cases of type 2 SCAD, intracoronary nitroglycerin should be administered to rule out vasospasm.^21^

Unlike atherosclerotic CAD, the optimal management of SCAD is undetermined because of lack of randomized trials comparing medical therapies and revascularization strategies. In addition, guideline‐directed medical therapies used for ACS have not been studied specifically in SCAD. Therefore, the current suggestions for the management of SCAD are based primarily on expert opinion from the observational series. The management of SCAD patients who are clinically stable without high‐risk features should be focused on medical therapy rather than coronary artery stent placement. Considerations for medical therapy include aspirin, beta blocker (±clopidogrel, ACE‐I/angiotensin receptor blockers [ARB], statins). These patients should be monitored in the hospital for 3 to 5 days.^21^ It is important to note that there are several areas of controversy in the medical management of SCAD as it relates to the use of statins and also the use of dual antiplatelet therapy (DAPT) in patients with SCAD. Generally speaking, the use of medications in patients with SCAD should be directed to the patient's non‐SCAD‐related indications rather than directed to the presence of SCAD itself. For instance, the use of ACE‐I should be used in patients with left ventricular dysfunction and the use of DAPT should be reserved for SCAD patients treated with coronary stent placement according to the ACS guidelines.^23^ For SCAD patients who are conservatively managed, it has been suggested that aspirin could be considered,^24^ but even this area requires further research as the optimal duration of this therapy remains unknown and this is especially relevant for these patients who are still having their menstrual cycles. The use of statins in SCAD is also controversial and generally, it is recommended that statins should only be used in SCAD patients who already have non‐SCAD related indications, such as a history of hyperlipidemia, atherosclerotic diseases, and/or diabetes with a goal low‐density lipoprotein (LDL) of <100 mg/dL.^23^ A small single‐center retrospective study had shown that statin use in SCAD patients was associated with a higher risk of recurrence of SCAD.^25^ Therefore, the use of statins should generally be avoided in patients with SCAD without other statin indications such as hyperlipidemia, diabetes, or atherosclerotic disease.

Coronary revascularization should be considered in patients with high‐risk features, such as left main dissection, ongoing/ recurrent ischemia or chest pain, ventricular tachycardia/fibrillation (VF/VT) and cardiogenic shock. Intra‐aortic balloon pump, extra‐corporeal membrane oxygenation, left ventricular assist device and Implantable cardioverter defibrillator is suggested in patients who are hemodynamically unstable with VF/VT and/or cardiogenic shock.^21^

Percutaneous coronary intervention (PCI) is challenging in SCAD patients because of the risk of propagation of the dissection or intramural hematoma risking further compromise to the coronary blood flow. There is also a risk of stent malapposition after resorption of the intramural hematoma (IMH) resulting in late stent thrombosis. The dissected segment of the vessels are often extensive requiring long stents and therefore increasing the risk of in‐stent restenosis. To address these challenges, it is suggested that PCI with stent placement is performed through the femoral approach with meticulous guide catheter manipulation and guided by OCT/IVUS to avoid stent malapposition and to ensure that the wire is in the true lumen of the vessel. Long stents should also be used to cover 5 to 10 mm of the proximal and distal edges of the IMH. Short stents placed at the proximal and distal edges of the dissection should be performed first followed by placement of a long stent in the middle.^21^ Bioabsorbable stents may also be considered as a temporary scaffold to avoid long‐term malapposition.^21^ Follow‐up OCT to assess for stent malapposition or the presence of uncovered struts prior to stopping aspirin and clopidogrel could be considered but is generally not recommended without a strong clinical indication as repeated invasive coronary angiography is not without increased risk in view of the fragility of the coronary arteries.^21^, ^23^

PCI should be considered in SCAD patients with isolated left main dissection and also recommended in patients with ongoing ischemia or chest pain, presence of VT/VF with or without cardiogenic shock if PCI is feasible. Coronary artery bypass graft surgery is suggested in SCAD if the LM dissection involves the LAD with or without left circumflex involvement and also if PCI is not feasible in the selected cases outlined above.^21^

Following a diagnosis of SCAD, it is very important to screen for predisposing arteriopathies associated with SCAD, such as fibromuscular dysplasia (FMD) with routine imaging of renal and iliac arteries during the baseline coronary angiogram with non‐selective abdominal and iliac aortograms. Alternatively, non‐invasive computed tomography angiography (CTA) of the renal and iliac artery may be considered although the sensitivity of this CTA is less than invasive angiography. CTA of the head and neck is also recommended to screen for cerebrovascular FMD and intracranial aneurysms which is seen in 14% to 25% of SCAD patients.^21^

Ongoing management of SCAD patients is important to decrease the risk of recurrence. Medical therapy includes adequate blood pressure control, use of a beta‐blocker could also be considered. .^21^ ACE‐I/ARB, DAPT, aspirin, and statins may be considered in cases where these are recommended for non‐SCAD‐related indications as discussed previously. Nitrates have not shown any beneficial effect in SCAD patients.^17^ However, it may be useful as an antianginal agent for patients with chest pain in whom coronary artery obstruction has been ruled out.^23^ Enrollment in cardiac rehabilitation is important with supervised graded exercise that avoids lifting weights greater than 20 lbs. To reduce arterial shear stress, target exercise heart rate (HR) to 50% to 70% of HR reserve on the basis of the entrance exercise treadmill test and systolic BP during exercise is limited to <130 mm Hg during this exercise program.^26^ Cardiac rehab has been associated with lower long‐term major adverse cardiac events (MACE) events.^21^ Psychosocial support is also important with counseling and peer group support.^16^ Recurrent pregnancy should be avoided in those where this occurred during pregnancy.^21^ Long‐term systemic estrogen and/or progesterone contraceptives or hormone replacement therapy should be avoided.^17^ Coronary CTA is not recommended as a first‐line imaging test for SCAD because of the lower spatial resolution. However, it may be useful as an alternative to invasive angiography to reassess the large proximal to mid‐arteries with SCAD to assess for arterial healing. Complete healing was observed by CTA at the median of 4 months post indexed event in >80% of cases.^21^

### 2.2 Myocardial ischemia with non‐obstructive coronary arteries

INOCA is more stable than MINOCA. The definition of INOCA in the absence of MI is dependent on the presence of three salient features which include (a) the presence of chronic stable symptoms suggestive of ischemic heart disease, such as chest discomfort that is anginal in nature or has atypical anginal features in terms of location, aggravating, and relieving factors, (b) objective evidence of myocardial ischemia either on EKG or myocardial imaging with nuclear perfusion, echocardiography, or cardiac magnetic resonance imaging (Cardiac MRI) during rest or stress (exercise, pharmacologic, or mental), and (c) the absence of flow‐limiting epicardial coronary lesion on invasive coronary angiography or coronary CTA with flow‐limiting disease defined as epicardial lesion with ≥50% stenosis and/or fractional flow reserve <0.8.^27^ INOCA is not a benign condition as INOCA has been shown through several prospective registries to be associated with a high risk of MACE, such as death, non‐fatal MI, and stroke as well increased hospitalization of heart failure and angina.^28^ INOCA is also associated with adverse effect in quality of life and functional status with decreased functional capacity. INOCA is also associated with recurrent visits to healthcare providers for recurring or persistent disabling symptoms..^28^ MSIMI usually presents as a form of INOCA rather than MINOCA as oftentimes patients will have features of ischemia in the absence of MI. MSIMI is an emerging presentation of CVD in women and is a poorly understood presentation and will therefore be further discussed in this document.

#### 2.2.1 Mental stress‐induced myocardial ischemia

There is a significant interplay between emotional stress and CVD, this interplay is most predominant in young women (<50 years of age) with CVD.^29^, ^30^, ^31^ MSIMI is more common in women with CVD (obstructive and non‐obstructive).^29^, ^30^ In addition, compared to men with CVD, women with CVD are more likely to have MSIMI and the presence of MSIMI confers a worse outcome in this group of women compared to their male counterparts with a 2‐fold higher risk of future cardiac events independent of physical stress‐induced ischemia in patient with stable CVD.^29^ It has also been shown that MSIMI post‐MI is more common in women 50 years or younger compared with similarly aged men.^32^, ^33^ However, these sex differences are not observed in post‐MI patients older than 50 years of age.^32^

**Figure 2:**
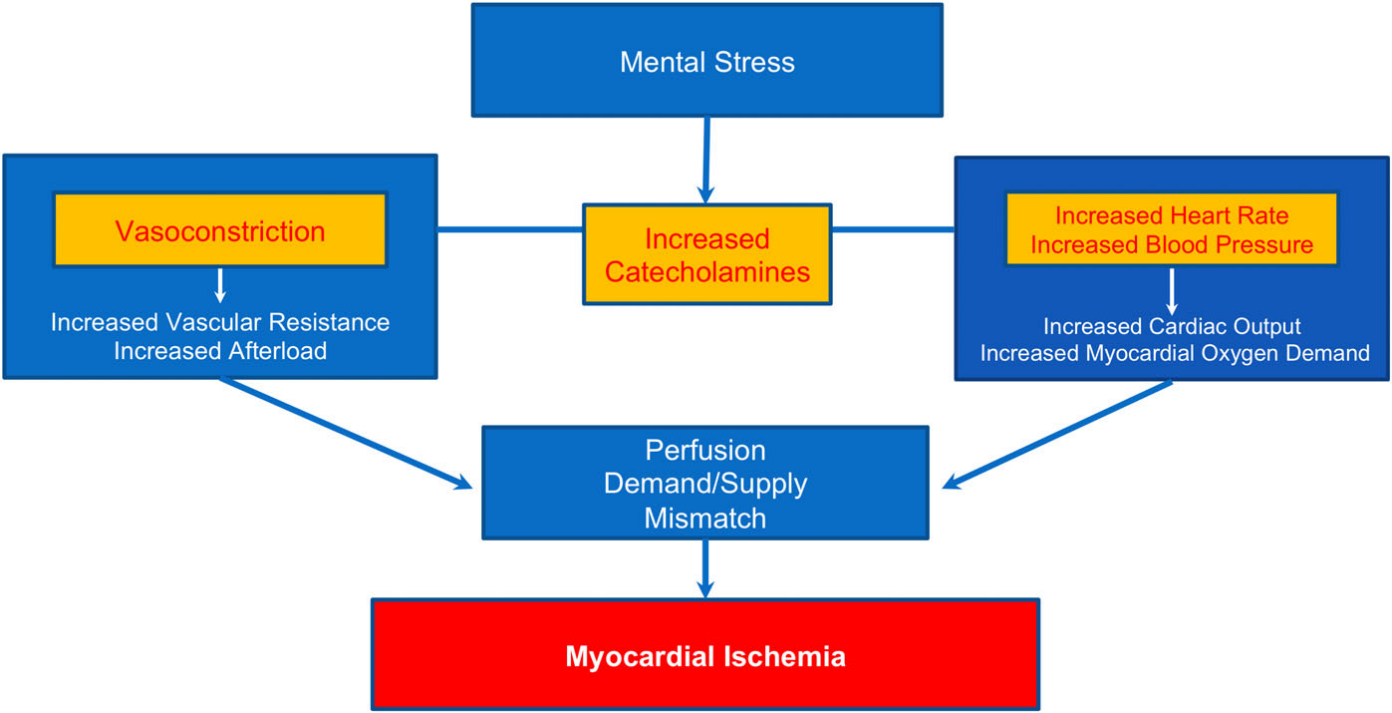
Pathophysiology of mental stress‐induced myocardial ischemia. Diagram outlining the underlying mechanism of mental stress‐induced myocardial ischemia

Microvascular dysfunction and peripheral vasoconstriction occurring with mental stress are thought to be the underlying cause of MSIMI among women but not in men.^29^ This is possibly associated with women's propensity towards having ischemia because of underlying microcirculation abnormalities^29^ Figure 2.

**Figure 3:**
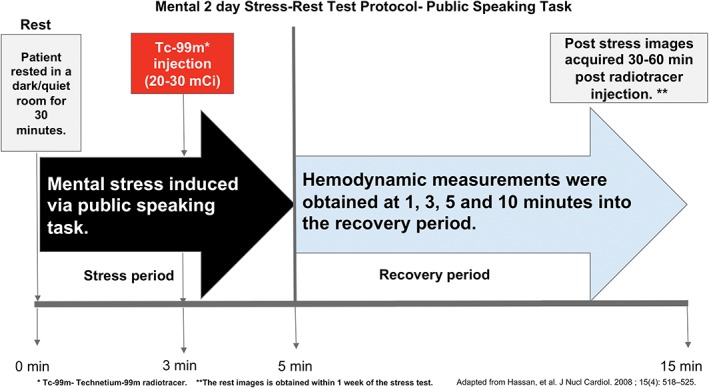
Mental stress test protocol—public speaking task. At rest the patient is initially rested in a dark and quiet room for 30 minutes while their heart rate (HR) and blood pressure are measured every 5 minutes using an electrocardiographic (ECG) monitor and automatic oscillometric device, respectively. Mental stress is induced through public speaking task on an assigned topic describing a stressful real life event. The speech is performed in front of a small audience, and the patient is given 2 minutes to prepare their speech and 3 minutes to speak. The patients are also told that their speech would be videotaped and later rated for content, quality and duration. Hemodynamic measurements are obtained at 1 minute intervals during the preparation and during the speech periods. Technetium‐99 m (Tc‐99 m) radiotracer injection (20‐30 mCi) is administered at 1 minute into the speech, which is 3 minutes into the stress period. Hemodynamic measurements were obtained at 1, 3, 5, and 10 minutes into the recovery period. Systolic blood pressure (SBP) and HR are used to calculate the double product (DP) value (DP = SBP × HR).Stress test is performed as a 2 day stress‐rest study. Post‐stress images acquired 30 to 60 minutes post‐radiotracer injection. The rest images is obtained within 1 week of the stress test

The pathology of mental stress‐induced ischemia in patient with ASCVD was identified with the use of myocardial radionuclide imaging almost 30 years ago by Rozanski et al. This study had identified the presence of ischemia with new wall motion abnormalities and/or decreased left ventricular ejection fraction (LVEF) defined as a decrease in more than 5% points from baseline LVEF on radionuclide ventriculography. Mental stress testing in that study was performed with a series of mental tasks including arithmetic, the Stroop color‐word task, simulated public speaking, and reading.^34^ Since then it has been shown that mental stress induces prolonged endothelial dysfunction indicating an important link between mental stress and ASCVD. .^35^ It has been more recently shown through the use of a novel mental stress protocol (Figure 3) that myocardial ischemia induced by mental stress occurs through a different mechanism of action when compared to ischemia induced by exercise or vasodilatory pharmacologic stress testing. Therefore, standard exercise or pharmacologic stress testing may not adequately assess the likelihood of occurrence or severity of MSIMI.^31^, ^36^ It has also been shown that MSIMI is more likely to occur in a single coronary artery territory distribution on SPECT imaging compared with exercise or pharmacologic stress‐induced ischemia.^36^

MSIMI is often missed on exercise or pharmacologic myocardial perfusion stress testing and often requires a mental stress test to be performed to illicit ischemia on myocardial perfusion imaging.^31^, ^37^ The degree of microvascular constriction is directly associated with the degree of MSIMI and this is independent of the angiographic burden of CAD.^31^

This concept of mental stress testing has also been studied in echocardiography. Impaired resting myocardial annular velocities on echocardiography have been found to be independently associated with mental‐stress‐induced ischemia in patients with coronary heart disease.^38^

In view of these findings of MSIMI in women with CVD, it is important that women with CVD are given the psychosocial support necessary to minimize life stressors. They should also be taught coping mechanisms so that they can change their reaction to these stressors as well. With regards to diagnostic testing, the studies outlined above suggest that mental stress testing is an underutilized resource. This underutilization may be as a result of lack of awareness and knowledge of mental stress testing protocols. The mental stress test protocol with a public speaking task outlined by Hassan et al.^36^ Figure 3 appears to be a reproducible protocol that could be adopted in labs in an effort to make the diagnosis of MSIMI. This should especially be considered in young women (<50 years of age) with ASCVD and angina in whom exercise or pharmacologic stress testing have been normal and new obstructive epicardial CAD has been ruled out.

## 3 CONCLUSION

CVD remains the leading cause of death among women in the United States and the mortality is particularly higher in younger women with this disease. There are several misunderstood emerging factors that are prevalent in young women, such as SCAD and MSIMI. It is important to recognize the presentations of these disease entities in young women to prevent misdiagnosis, missed diagnosis as well as mismanagement of these patients to improve their clinical outcomes.

## CONFLICT OF INTEREST

The authors declare no potential conflict of interests.
